# Effects of Different Coatings, Primers, and Additives on Corrosion of Steel Rebars

**DOI:** 10.3390/polym15061422

**Published:** 2023-03-13

**Authors:** Alireza Afshar, Soheil Jahandari, Haleh Rasekh, Aida Rahmani, Mohammad Saberian

**Affiliations:** 1 Department of Civil and Environmental Engineering, George Mason University, Fairfax, VA 22030, USA; aafshar2@gmu.edu; 2 Centre for Infrastructure Engineering, Western Sydney University, Penrith, NSW 2751, Australia or soheil@chemconcrete.com.au (S.J.); or aida@chemconcrete.com.au (A.R.); 3 Chem Concrete Pty Ltd., Seven Hills, NSW 2147, Australia; 4 School of Civil and Environmental Engineering, University of Technology Sydney, Sydney, NSW 2007, Australia; 5 School of Engineering, RMIT University, Melbourne, VIC 3000, Australia; mohammad.boroujeni@rmit.edu.au

**Keywords:** accelerated corrosion, coating systems, rebars, inhibitors, pozzolanic materials, pullout test of steel–concrete bond joints

## Abstract

In this research, methods of increasing the corrosion resistance of reinforced concrete were experimentally investigated. The study used silica fume and fly ash at optimized percentages of 10 and 25% by cement weight, polypropylene fibers at a ratio of 2.5% by volume of concrete, and a commercial corrosion inhibitor, 2-dimethylaminoethanol (Ferrogard 901), at 3% by cement weight. The corrosion resistance of three types of reinforcements, mild steel (STt37), AISI 304 stainless steel, and AISI 316 stainless steel, was investigated. The effects of various coatings, including hot-dip galvanizing, alkyd-based primer, zinc-rich epoxy primer, alkyd top coating, polyamide epoxy top coating, polyamide epoxy primer, polyurethane coatings, a double layer of alkyd primer and alkyd top coating, and a double layer of epoxy primer and alkyd top coating, were evaluated on the reinforcement surface. The corrosion rate of the reinforced concrete was determined through results of accelerated corrosion and pullout tests of steel-concrete bond joints and stereographic microscope images. The samples containing pozzolanic materials, the corrosion inhibitor, and a combination of the two showed significant improvement in corrosion resistance by 7.0, 11.4, and 11.9 times, respectively, compared to the control samples. The corrosion rate of mild steel, AISI 304, and AISI 316 decreased by 1.4, 2.4, and 2.9 times, respectively, compared to the control sample; however, the presence of polypropylene fibers reduced the corrosion resistance by 2.4 times compared to the control.

## 1. Introduction

One of the main reasons for the decreasing durability of reinforced concrete is the corrosion of steel reinforcement in concrete structures. Reinforcement corrosion in concrete is an electrochemical process which consists of anode, cathode, and an electrolyte which is the aqueous soluble electrolyte inside the concrete cavities [1,2,3]. The potential difference between the anodic and cathodic points is the corrosion electromotive force of the steel.

The corrosion of reinforcement in existing structures affects bond behavior, structural performance, and fatigue damage [4,5,6]. Previous research has shown that degradation of the bond between steel bars and concrete can have an adverse effect on the load-carrying capacity of structural members under bending conditions [7,8]. In addition, Cavaco et al. [9] reported that more extensive corrosion can have an impact on the tension force and bond behavior between concrete and steel. They found that even small losses in area can cause complete debonding between concrete and steel, rendering the reinforcement ineffective. Furthermore, for corrosion levels close to 30%, the stress in steel is negligible, and the residual capacity is provided by the non-corroded inner reinforcement layers. Lin et al. [10] reported that more investigation is required to investigate the bond behavior of corroded steel subjected to cyclic loading.

In general, the methods of increasing the corrosion resistance of reinforced concrete structures can be divided into several general categories; (1) incorporating additives in the concrete mix design, (2) changing of steel reinforcement, (3) adding corrosion inhibitors to concrete mix design, (4) using a coating on steel reinforcement in concrete, (5) considering cathodic protection with direct current and sacrificing anodes, and (6) adopting electrochemical extraction of chloride ion to reduce corrosion [11,12,13,14].

Various additives have been suggested by researchers to enhance the durability properties of concrete [15,16,17]. Among these additives, fly ash is a commonly recommended additive concrete in coastal environments [18,19,20,21,22,23,24]. In this regard, Thomas and Bamforth [25] presented a model for chloride ion penetration in concrete. They showed that the chloride ion diffusion coefficient decreased and the service life of concrete increased with an increasing fly ash content. Fu et al. [26] found that increasing the water/binder ratio of cementitious materials led to higher binding capacity and lower chloride resistance, regardless of binder type. Up to 70% gradual replacement of fly ash improved the chloride resistance and binding capacity of cementitious binders. Byfors [27] found that adding silica fume or fly ash to ordinary Portland cement paste reduced the diffusion rate of chloride.

According to Malhotra‘s research [28], the use of fly ash, superplasticizer, and micro-silica improves the durability and provides high-performance concrete. Montes et al. [29] studied the effect of various percentages of fly ash, along with sodium nitrite inhibitor, in cracked concrete and found that the optimum amount was 20% by weight of cement. Sekulic et al. [30] compared the strength of concrete containing 20% fly ash to that of Portland cement concrete and found that using mechanically activated fly ash increased the compressive strength by 57.6% after a 28-day curing period. Another study [31] reported a 26% increase in compressive strength after one year of curing with the use of fly ash. Montemor et al. [32,33] evaluated the impact of chloride ion penetration and corrosion on rebars in concrete containing 30% fly ash using electrochemical tests, such as impedance and open-circuit potential tests. Many other studies have evaluated the optimum amount of fly ash for improving the strength and corrosion resistance of reinforced concrete, with the majority reporting an optimum amount of 20–30% of cement weight [34,35,36,37,38,39,40,41,42].

Dotto et al. [43] studied the impact of various percentages of silica fume on the durability of concrete. They found that adding 12% of silica fume increased the concrete strength by five times (due to the pozzolanic reaction) and reduced the pH of the solution in the concrete pores around the reinforcement, which was a result of the reaction with concrete lime and reduced passive current caused by the oxide layer on the reinforcement surface. The addition of silica to concrete modifies its microstructure by increasing its density and uniformity [44,45]. Matte and Moranville [46] showed that concrete containing 10% silica was 1.5 times stronger than non-additive concrete after 90 days of curing. Videm [47] conducted electrochemical tests and reported that the use of silica enhances the corrosion resistance of reinforcements in concrete and its positive effect on the concrete microstructure is greater than its negative impact on the pH of the solution in the concrete pores around the reinforcement.

Another effective additive for concrete is polypropylene fibers. These fibers have a melting point of approximately 165 °C and are chemically neutral, making them resistant to many chemicals. Their hydrophobic surface prevents them from absorbing the cement paste. Thus, the addition of these fibers does not affect the water content in concrete. With shear stresses in the range of 45–65 ksi, these fibers are used in multi-strand form. Optimal use of fibers delays crack propagation, reducing steel rebar corrosion and improving concrete’s compressive strength [48,49,50]. According to another study [51], the initiation of cracks due to steel corrosion in concrete containing polypropylene fibers occurs over longer periods compared to concrete with nylon fibers and without fibers.

By increasing the resistance of rebars, their resistance to corrosion can be improved. According to the Norwegian Urban Research Association report, the corrosion resistance of AISI 304 and AISI 316 steels is 19 and 25 times higher than that of mild steel, respectively [52]. Addari et al. [53] found that corrosion-resistant steels, such as AISI 304 and AISI 316, are much more resistant to pitting corrosion in a simulated concrete solution than plain carbon steel. Research [54] showed that the critical concentration of chloride required to break the passive layer on AISI 304 and AISI 316 steels is about 10 times higher than that of carbon steel. Kelestemur and Yildiz [55] studied the effects of different dual-phase heat treatments on the corrosion resistance of reinforcements used in concrete structures and found that the proposed heat treatment could increase the strength of the reinforcement in concrete.

The use of corrosion inhibitors is aimed at preventing the reaction of chloride ions with the reinforcement surface and increasing the time it takes for chloride ions to penetrate the concrete. The ideal inhibitor is a chemical compound that can prevent corrosion of the reinforcement without harming the concrete properties. 2-Dimethylaminoethanol, sold under the brand Ferrogard 901 and belonging to the Amino Alcohol Inhibitors group, has been studied for its inhibitory mechanism. Jamil et al. [56,57] reported the mechanism of corrosion as anodic and a reduction in the corrosion potential, while Gaidis [58] reported that the mechanism of corrosion is by attacking cathodic activity and blocking sites where oxygen picks up electrons, thus slowing the oxygen resuscitation reaction. The molecular structure of this inhibitor is (CH3)2N-CH2-CH2-OH [59], which is absorbed by the reinforcement surface and forms a layer 2–10 nm thick on the surface [60].

One of the most effective ways to prevent steel-reinforced concrete from corroding is to use protective coatings on the reinforcement surface. Galvanized coating, made of multiple layers of iron-zinc alloys that are metallurgically bonded to the underlying steel, is a commonly used corrosion-resistant coating [61]. Another option is painting the metal surface, though multilayer paint systems are necessary in highly corrosive environments where the concentration of chloride ions on the steel exceeds a certain threshold [62]. Alkyd resin-based paints, which have excellent water resistance and can be used as a primer and middle layer for topcoats, are dried with oxygen [63]. Epoxy resin-based paints, which are resistant to chemicals, are another option, though they are susceptible to concentrated oxidizing materials in the long term [63,64]. Resin-based polyurethane coatings, up to 400 microns thick, have excellent physical and chemical properties and can withstand abrasions, scratches, impacts, and corrosive environments in coastal and industrial areas [63]. EN ISO 12944-5 defines various types of commonly used protective paints and coatings for structures [65].

It is likely that using inhibitors and additives in concrete, as well as applying protective coatings on reinforcements, can significantly improve the corrosion resistance of reinforcements. However, the combined effects of these methods have not been thoroughly studied. In this study, five different concrete mixes were prepared, using Ferrogard 901 inhibitors, silica fume, fly ash, polypropylene fibers, and combinations of these additives, to examine their impact on the corrosion rate of concrete. Three types of reinforcements were used: st37 (mild steel), AISI 304 (stainless steel), and AISI 316 (stainless steel). Nine different coating systems were applied on the reinforcement surface to increase corrosion resistance: hot-dip galvanized coating, alkyd-based primer, zinc-rich epoxy primer, alkyd top coating, polyamide epoxy top coating, polyamide epoxy primer, polyurethane coatings, double layer of alkyd primer and alkyd top coating, and a double layer of epoxy primer and alkyd top coating. An accelerated corrosion test (ACT) and a pullout test of the reinforcements were conducted, and stereographic microscope images were used to assess the corrosion rate of each system and compare the effectiveness of all the protective techniques quantitatively.

## 2. Research Significance

This research offers a comprehensive understanding of how to enhance the corrosion resistance of steel-reinforced concrete. By examining various concrete mixes, additives, reinforcing bars, coating systems, and primers, it provides a valuable source of information on how to select the most appropriate mixture, rebar, coating, and primer to meet specific requirements. A systematic study is carried out to evaluate the combined or individual impact of these systems and to compare their performance, leading to a unique conclusion on the best approach to increase corrosion resistance.

## 3. Materials and Methods

### 3.1. Materials

In this study, three types of alloys were used, including mild steel (st37) and two different grades of stainless steel (AISI 316 and AISI 304) based on the American Iron and Steel Institute (AISI, Washington, DC, USA). The chemical composition of the alloys is shown in Table 1. Portland cement (type 2, Tehran, Tehran, Iran) was utilized in the concrete mixes and Table 2 displays the chemical properties of Portland cement, silica fume (Tehran, Tehran, Iran), and class F fly ash (Tehran, Tehran, Iran), which were also included as pozzolanic and polymeric additives. The concrete was reinforced with polypropylene fibers with a diameter of 30 µm, length of 12 mm, density of 0.91 kg/m^3^, and tensile strength of 350 MPa. Additionally, FerroGard 901 inhibitor from the SIKA (Tehran, Tehran, Iran) was added to the concrete mix as an inhibitory additive. Table 3 provides the details of the FerroGard 901 inhibitor. Finally, twice-washed granite aggregates, sourced from Ekhtiarabad Query, Kerman, Iran, with a grain size ranging from 4.5 to 9 mm and tap water were used in the concrete mixes.

### 3.2. Mix Designs of Concrete Samples

In this study, five different concrete mixes were prepared. The first mix was prepared according to the ACI recommendations with a water-to-cement ratio of 0.40 and exposed aggregate and cement to corrosive environments. To evaluate the effect of the FerroGard 901 inhibitor on corrosion resistance, the second mix included the inhibitor at a 3% weight ratio of cement. The third mix was created by adding fly ash (at 25% weight ratio of cement) and silica fume (at 10% weight ratio of cement) to the first mix, with a water-to-binders ratio of 0.40. The fourth mix used 5% fly ash, 10% silica fume, and the FerroGard 901 inhibitor simultaneously. The fifth mix evaluated the influence of polypropylene fibers, at an optimum percentage of 2.5% by volume of concrete.

In each mix, sand and aggregate were mixed for two minutes, followed by the addition of cement in one minute. Then, water was slowly added to the mix over two minutes, along with the superplasticizer and FerroGard 901 inhibitor. The maximum time allowed for filling the mold was half an hour, after which the concrete was vibrated with a frequency of 30 Hz using a vibration table and poured into the molds in two stages with a 20-s interval. The concrete samples were removed from the molds after 24 h and immersed in 27 °C fresh water for 14 days to complete the hydration reaction.

Table 4 shows the 12 different reinforcement designs used in the 5 mix designs, resulting in 60 different combinations in total. Three and two samples were tested for the non-coated and coated reinforcement specimens, respectively, for data repeatability. The reinforcement design, for example, mild steel rebar with a zinc-rich epoxy primer in the third mix design, is represented as FS-MS-ZP, meaning “mix design-steel rebar type-coating/primer.”

### 3.3. Sample Preparation

The 10 mm diameter steel reinforcement was cut into 120 mm length rebars. The rebars were then treated in a Sonica-1200M sonicator (Tehran, Tehran, Iran), with a 0.005% urotrop in inhibitor in a hydrochloric acid solution to remove iron rust (for st37 reinforcements). After being treated, the rebars were washed with tap water and sanded with 60 to 600 sandpapers to achieve the best surface quality for painting. The paint thickness was selected based on the manufacturer’s recommendation for corrosive environments, as shown in Table 5. The electrochemical test was conducted using cylindrical PVC molds with a diameter of 86 mm and a height of 120 mm. A 37.7 cm^2^ cross-sectional area of the 120 mm steel reinforcement was used for corrosion resistance testing, with the reinforcement placed in the middle of the cylindrical mold. The molds were carefully sealed to prevent any water leakage.

### 3.4. Experiments

#### 3.4.1. Accelerated Corrosion Test

**Figure 1:**
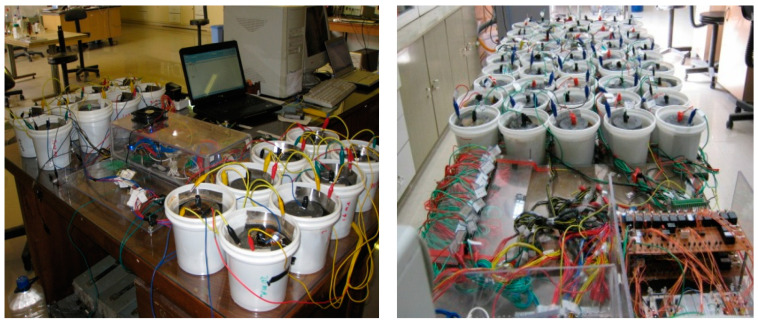
The accelerated corrosion test which measured and displayed data in a corrosion measuring device.

In this study, the accelerated corrosion test with anodic potential was conducted according to ASTM-G 109, West Conshohocken, PA, USA (Figure 1). This test, also known as the accelerated chloride ion penetration test, was performed to calculate the corrosion rate and investigate the structure life of concrete, which had low porosity and was coated with systems. The anodic potential was applied, and the anodic flow was recorded between the reinforcement and the auxiliary electrode. A direct voltage source, a concrete specimen immersed in a 3.5% NaCl solution according to ASTM G22-99, West Conshohocken, PA, USA [66], two U-shaped AISI 304 plates (West Conshohocken, PA, USA) around the concrete specimen (cathode), and a measuring device to record the corrosion rates were used. The steel reinforcement served as the anode and was connected to a positive voltage source, while the steel plates were the cathode and connected to the negative voltage (auxiliary electrode). A constant 32 V was applied until the specimens were damaged, indicated by the propagation of a 0.5 mm crack.

Four power supplies were used simultaneously with a maximum voltage of 32.5 V and a current of 4 amperes. The device had 64 inputs, each connected to an auxiliary electrode, and 8 microcontrollers were programmed to measure and record the flow at 1 h intervals. The software displayed the changes in corrosion current for each specimen at each time interval. The positive pole of the sources was connected to a board with 64 outputs, and each output was connected to a sample of steel reinforcement. The current varied in the range of 100 µA to 500 mA due to changes in concrete strength after the application of anodic potential.

#### 3.4.2. Pullout Test of Steel–Concrete Bond Joints

**Figure 2:**
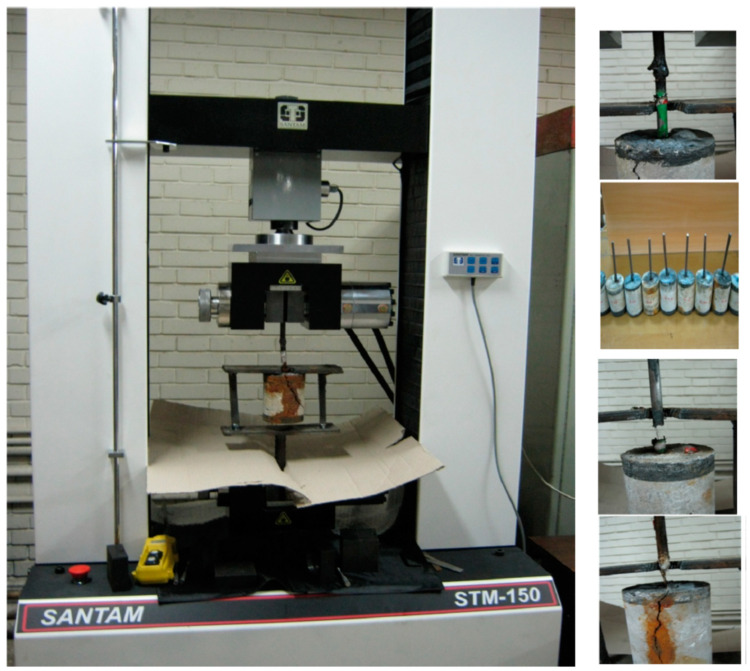
Pullout of steel–concrete bond joints test apparatus.

The pullout test of steel–concrete bond joints was carried out using a 150-ton capacity tensile testing machine manufactured by Santam (Tehran, Iran), with a loading speed of 10 mm/minute. A retainer was made to securely hold the concrete specimen in the center of the testing device and apply concentric forces to it, reducing any potential twisting between the concrete and steel reinforcement (as shown in Figure 2). The machine was first calibrated using a low-force application on the specimen, before the test was carried out.

## 4. Results and Discussions

### 4.1. Accelerated Corrosion

The results of the accelerated corrosion test for each of the five mix designs were analyzed individually. The sample was deemed to have failed when a crack with a width of at least 0.5 cm appeared due to the expansion of corroded steel [67]. Studies of accelerated corrosion tests with a constant anodic potential have shown that the time of change in the curvature before the peak of the maximum flow curve corresponds to the time of concrete cracking [68]. The variations in flow in the three amplitude ranges of milliampere, microampere, and nanoampere are variables. Since it is not possible to represent both milliampere and microampere for comparison in each mix design, samples with high corrosion current density are displayed on the curve in milliamperes and samples with low corrosion current density are shown on the curve in microamperes. The corrosion current density was calculated by dividing the obtained current by the surface area of the reinforced concrete. All graphs plot the current density against the elapsed time (hours).

#### 4.1.1. Accelerated Corrosion Evaluation in the First Mix Design

**Figure 3:**
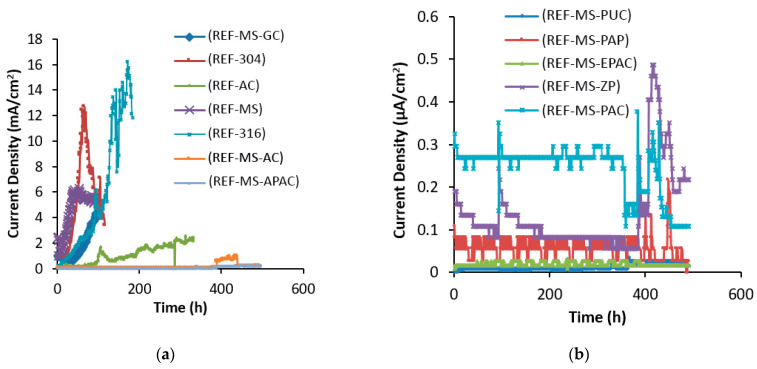
Corrosion current densities over time for different coating systems; (**a**) milliampere range, (**b**) microampere range in the first mix design.

Figure 3 illustrates the changes in corrosion current densities over time for various coating systems. The non-coated steel reinforcement sample degraded after 26 h when a 32 V anodic potential was applied, and rebars of AISI 304, galvanized steel, AISI 316, and the alkyd primer system were destroyed. However, the corrosion current density of the alkyd top coating increased but the sample was not degraded during the 500 h test period. The corrosion of embedded steel causes high stress on concrete, which can reach up to 450 MPa and destroy the concrete within 24 h.

The protection systems in the first mix design can be divided into two categories:

1. This group consisted of systems that either suffered damage after 500 h or were highly prone to damage due to the high current flowing through the applied layer. The alkyd top coating, alkyd primer, alkyd top coating and double layers of alkyd primer, AISI 304, AISI 316, and galvanized steel were included in this group according to the curves in Figure 3a. In the first mix design, chloride ions from the 3.5% NaCl solution quickly penetrated into the porous concrete due to the lack of additives in the mix and reached the critical level (0.4% of the cement weight in the concrete with mild steel reinforcement rebar), causing damage to the protection layer [69,70]. Tuutti’s proposed model [1] divides the corrosion of the reinforcement into three stages: impaction, breaking of the upper layer, and expansion of corrosion in the reinforcement leading to the destruction of the structure. The reported numbers, which correspond to the third stage, represent the progress of corrosion. The threshold chloride ion concentration on the reinforcement surface varies with each method, so the failure of the protective layer will also vary. Research on non-coated steel rebar in the non-additive mix design shows that the breakdown of the oxide top layer occurs when the corrosion current density exceeds 0.3 μA/cm^2^ [71]. As seen in Figure 3a, failure occurred during the early hours of anodic potential application. Figure 3b shows that the corrosion current density of all specimens was below the critical range, so it can be concluded that no corrosion occurred.
2. This group included systems that are resistant to corrosion and did not experience the third stage of corrosion expansion. The chloride ion threshold for destroying coatings was not reported in the sources, so weight loss was calculated using Faraday’s law and the corrosion current density, and the corrosion rate was used as a criterion for comparing these coatings to the measured corrosion current density. Debaiky et al. [72] and Auyeung et al. [73] observed a concurrence between the weight loss and measured anodic flow test data. Table 6 shows the demolition time and corrosion rate of the protective systems in the first mix design.

The corrosion resistance of steel reinforcement increased with the increase in polarization resistance of the coating. The cracking time of AISI 304 and AISI 316 stainless steels was found to be 1.96 and 4.96 times that of non-coated steel rebars, respectively. This suggests that as the resistance of the coating layer increased, the corrosion resistance also increased in proportion. The chloride ion degradation threshold was higher for stainless steels than for non-coated steel reinforcement. The use of AISI 316 and AISI 304 rebars increased the structural durability by up to 5 and 2 times, respectively, in the first mix design. However, the corrosion rate of the AISI 316 rebar was 158 mpy under the current conditions. It should be noted that these values are relative and the potential difference of 32 V will not occur in the structure. The corrosion rate of galvanized steel was 42 mpy, as shown in Table 6, and according to Cheng et al. [74], the corrosion rate of galvanized steel with a potential of 30 V was reported to be 37 mpy. This difference can be attributed to the difference in the applied potential and concrete mix design. The galvanized steel provided protection by forming calcium zinc salts and reinforcing the steel rebar, which delayed corrosion. On the other hand, galvanized steel with a solubilization mechanism of zinc instead of steel (cathodic protection with sacrificing local anodes) resulted in increased corrosion resistance of the steel. In the first mix design, the adoption of galvanized steel increased the corrosion resistance of steel by 2.57 times, which was greater than that of AISI 304 rebar and lower than that of AISI 316 rebar. Hot-dip galvanizing was found to be a more economically and operationally justifiable process than other protection methods.

The use of alkyd primer increased the corrosion resistance by up to 3.8 times, but its use was not justifiable due to the cost and risk of damage. It is important to ensure that the primer is not damaged before it is installed in concrete. The use of primer for protecting steel reinforcement is not recommended due to the cost and risk of damage, despite its high protection strength. When steel reinforcement with a damaged surface, such as from corrosion or preinstallation, is enclosed in concrete, the anode will corrode due to the large cathode surface.

Using paint with high adhesion, durability, and strength can provide good protection against corrosion for the reinforcement. Polyurethane, polyamide epoxy, and zinc-rich epoxy top coatings had good adhesion to the reinforcement, unlike alkyd coating. By comparing the results in Figure 3, it can be seen that the current density of all these coatings varied in the microampere range. Polyurethane top coatings, alkyd epoxy polyamide coatings, epoxy polyamide coatings, zinc-rich epoxy coatings, and epoxy polyamide coatings had the lowest corrosion current density. Zinc was also found to be a reliable galvanizing system with a corrosion rate of 0.002 mpy and a galvanizing protection mechanism.

#### 4.1.2. Accelerated Corrosion Evaluation in the Second Mix Design

**Figure 4:**
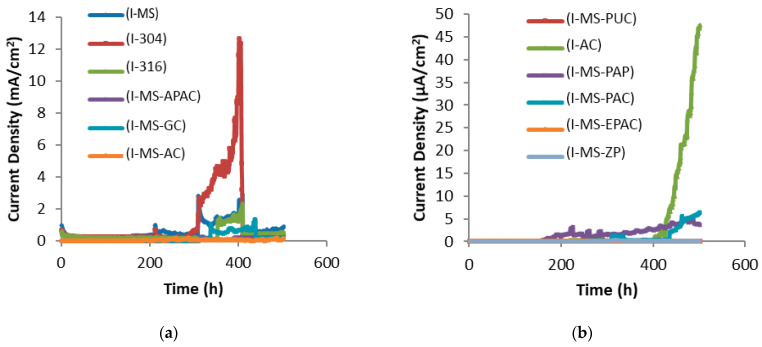
Corrosion current density over time for different coating systems; (**a**) milliampere range (**b**) microampere range in the second mix design.

The Ferrogard 901 inhibitor was utilized in the second mix design, which resulted in a delay in cracking time by reducing the corrosion rate. The cracking time of various coating systems in the second mix design is shown in Figure 4, and summarized in Table 7. The systems, such as AISI 304, AISI 316, galvanized steel, and double layers of alkyd primer and alkyd top coatings, were categorized in the high current range. The presence of the Ferrogard 901 inhibitor increased the threshold chloride level and delayed steel degradation. As can be seen when comparing Table 6 and Table 7, the addition of the Ferrogard 901 inhibitor caused the cracking time of concrete to be delayed by 11.42, 4.80, 6.09, and 1.20 times for mild steel, galvanized steel, AISI 304 rebar, and AISI 316 rebar, respectively. The alkyd primer was not destroyed in this mix design, but the Ferrogard 901 inhibitor changed the corrosion resistance parameters through adsorption of the top layer of the reinforcing layer, which requires further investigation through adsorption tests. The Ferrogard 901 inhibitor also resulted in a decrease in current for microampere extension coatings, reflecting the formation of a reinforced film on the reinforcement. The results showed that the inhibitor reduced the corrosion rate by up to three times in mild steel reinforcement, thus improving both the concrete properties and the formation of a layer on the steel reinforcement surface. This reduction was achieved by slowing down the diffusion mechanism and the reaction of chloride ions with the reinforcement surface.

#### 4.1.3. Accelerated Corrosion Evaluation in the Third Mix Design

**Figure 5:**
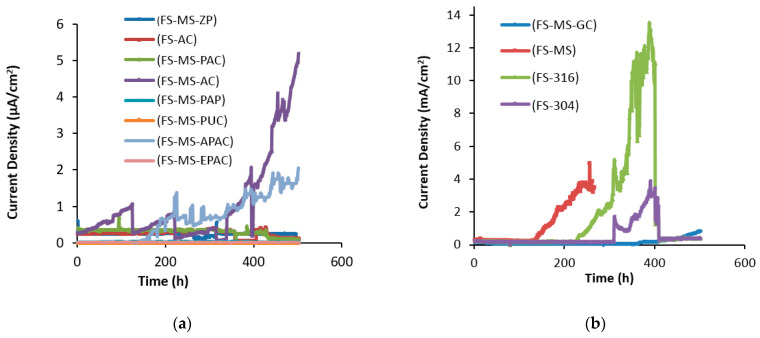
Corrosion current density over time for different coating systems; (**a**) microampere range, (**b**) milliampere range in the third mix design.

In the third mix design, 25% fly ash and 10% silica fume were adopted by weight of cement. The correlation between corrosion current density and time for different coating systems and steel reinforcement types is displayed in Figure 5. Figure 5b shows that non-coated mild steel, galvanized steel, AISI 304, and AISI 316 rebars were in the microampere current range in the mix design, while the other protection methods displayed in Figure 5a were in the microampere range.

Table 8 displays the destruction time and corrosion rate for all samples. The corrosion resistance of non-coated steel reinforcement, galvanized steel, AISI 304 rebar, and AISI 316 rebar in the third mix design increased by 7.03, 15.70, 11.92, and 12.15 times, respectively, compared to the non-coated reinforcement in the first mix design. The rest of the coating methods, as shown in Figure 5a, shifted to lower microampere currents than in the first mix design.

According to Kayali and Zhu’s research [4], adding 10% silica fume to the mix design increased the corrosion resistance of steel by seven times. However, in the current research, incorporating 10% silica fume and 25% fly ash only decreased the corrosion rate by 1.47 times compared to the first mix design, and by more than 2 times compared to the second mix design. Therefore, using the Ferrogard 901 inhibitor is more effective in decreasing the corrosion rate than using pozzolanic additives like silica fume and fly ash. Further investigation is needed to interpret the reasons for the increasing or decreasing resistance, due to the different surface reactions of the systems used. According to Table 8, the corrosion rate of the coating systems used was very low, with the lowest rate of 0.0001 mpy related to the alkyd top coating and 0.00021 mpy related to the polyurethane top coating.

#### 4.1.4. Accelerated Corrosion Evaluation in the Fourth Mix Design

**Figure 6:**
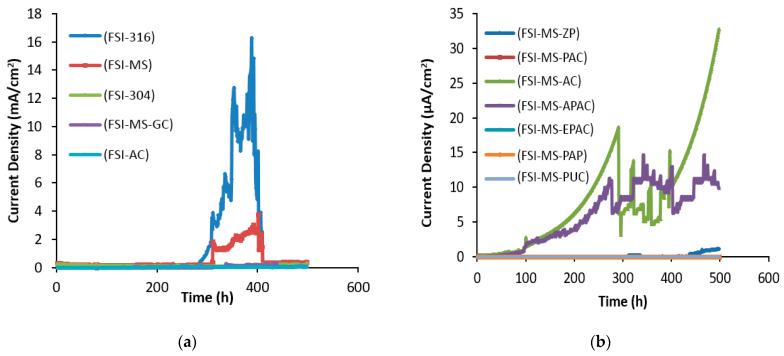
Corrosion current density over time for different coating systems; (**a**) milliampere range, (**b**) microampere range in the fourth mix design.

Figure 6 shows the corrosion current densities of various protection systems in the fourth concrete mix design, which incorporated 10% silica fume, 25% fly ash, and 3% Ferrogard 901 inhibitor by weight of cement. The results of these tests are significant as they quantified the increase in corrosion resistance of steel reinforcement in the presence of both the inhibitor and pozzolanic materials, and compared it with the first mix design. It can be seen that the corrosion resistance of non-coated mild steel reinforcement in the fourth mix design increased 11.92 times and was destroyed after 310 h, compared to the first mix design. The AISI 304 rebar was also destroyed after 354 h, showing a seven-times decrease in the corrosion rate.

Figure 6a shows that the current densities in the fourth mix design shifted to lower values. The presence of the Ferrogard 901 inhibitor, with a pH of approximately 10 (lower than the concrete pH of approximately 13), increased the stability of epoxy primers. The reduced alkalinity can help improve dye stability in concrete. Al-Mehthel et al.’s research [5] on silica-containing concrete with an organic inhibitor showed that the use of the inhibitor increases the cracking time in concrete from 36 to 89 h. In this study, the fourth mixing design delayed concrete degradation from 26 h in the first mix design to 310 h (11.92 times).

Table 9 displays the destruction time and corrosion rate of the systems used in the fourth mix design. The lowest corrosion rate for the primer-coating system was related to the double layer of epoxy primer and alkyd top coating, with a corrosion rate of 0.000014. The results showed a two-times increase in corrosion resistance by incorporating alkyd surface top coating to the epoxy primer.

#### 4.1.5. Accelerated Corrosion Evaluation in the Fifth Mix Design

**Figure 7:**
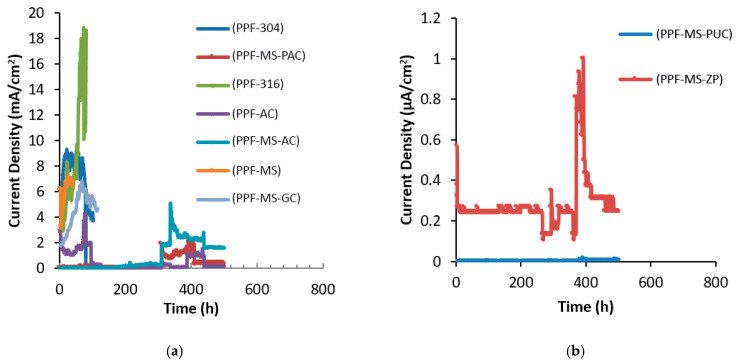
Corrosion current density over time for different coating systems; (**a**) milliampere range, (**b**) microampere range in the fifth mix design.

In the fifth mix design, polypropylene fibers at 2.5% volumetric concrete were used. Figure 7 demonstrates the corrosion current density curves over time. In this mix design, which experienced a very high corrosion rate, the efficiency of the coatings can be more accurately estimated. In the curves of Figure 7a, it can be seen that galvanized steel specimens, AISI 304 rebar, AISI 316 rebar, alkyd primer, alkyd top coating, non-coated mild steel rebar, and polyamide epoxy top coating were in the range of milliampere currents; however, in previous mix designs, the current flows were much lower. The destruction time and corrosion rate of each coating are shown in Table 10.

Although the coatings of zinc-rich epoxy primer and polyurethane epoxy top coating were in the range of microamperes, they are distinguished by their protection mechanism. In the zinc-rich epoxy primer, there are two mechanisms related to the protection. The first mechanism is the creation of a barrier and adhesive protective layer based on the dye adhesion test, and the second is the cathodic protection of the zinc particles in the coating. The corrosion rate remains low as long as zinc exists in the coating [75]. On the other hand, thanks to the relatively higher conductivity of zinc-rich epoxy primer compared to the polyurethane epoxy top coating, the concentration of the current under the color layer (in the coating and reinforcement interface) was not observed. The debonding, thanks to the dissolving layer in zinc-rich epoxy primer, was not as strong as in the polyurethane epoxy top coating. Evidence of this assertion was found when the reinforcement was removed from the concrete. However, the polyurethane epoxy top coating system is the only barrier against harmful chloride ions [76].

As Table 10 depicts, the destruction time of non-coated steel, galvanized, AISI 304 rebar, AISI 316 rebar, and alkyd primer was shorter than that of similar samples in the first mix design, and the current density values in milliampere curves were higher (higher density of corrosion currents). The other modes are not discussed here, thanks to the similarity of the mechanism of epoxy coatings in this case. The cracking time of concrete in the concrete sample with non-coated reinforcement was reduced by 2.7 times.

Therefore, the use of high-strength polypropylene fibers in concrete is not recommended for improving concrete’s corrosion resistance. Using these fibers increased the corrosion by up to three times, which could be attributed to the following two factors:

Use of polypropylene fibers resulted in fast adhesion, low usability, and increased viscosity of mortar.

When using the vibration table, thanks to the high viscosity of fresh concrete, it was very difficult to fill the molds and the time required for removing the entrapped air bubbles from the concrete was not available. Therefore, concrete became more porous thanks to the entrapped air, and the corrosive ion transfer pathways increased and reached the reinforcement surface; therefore, corrosion occurred in a much shorter time.

#### 4.1.6. Comparison between Non-Coated Mild Steel Reinforcement in the Five Mix Designs Using Accelerated Corrosion Testing

**Figure 8:**
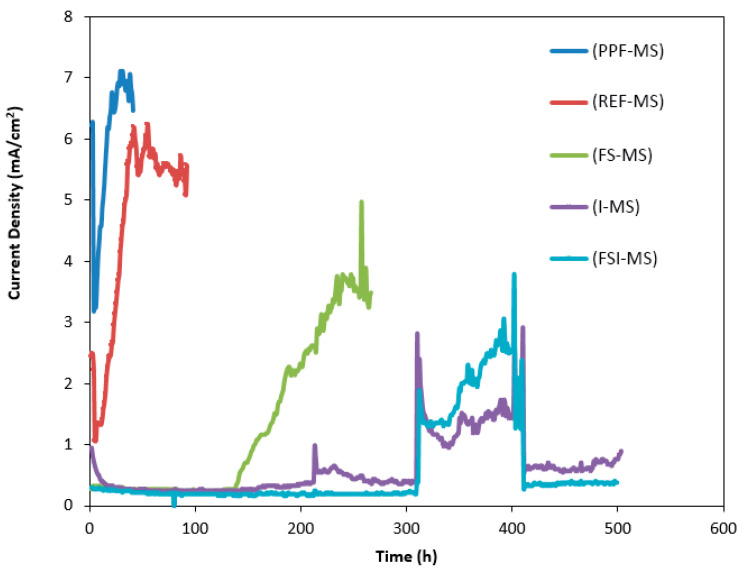
Corrosion current density versus time for non-coated mild steel reinforcement in the five mix designs.

The reasons for increasing or decreasing the corrosion resistance of reinforced concrete in each mix design were investigated. Figure 8 shows the corrosion current density versus time for non-coated reinforcement in each mix design. According to Figure 8, the fourth mix design containing 10% silica fume, 25% fly ash, and 3% Ferrogard 901 inhibitor was the best choice, followed by the second and third mix designs; however, the fifth mix design incorporating polypropylene fibers was the most inappropriate. A quantitative comparison of reinforcement elimination times for all five mix designs is shown in Table 11. Therefore, by considering the accelerated corrosion testing, the fourth mix design could be recommended as the most suitable mix design for corrosion control purposes. 

### 4.2. Pullout Test of Steel–Concrete Bond Joints 

If the required shear stress is less than the critical threshold stress calculated inside the concrete (the interface of reinforcement and concrete), the rebar does not satisfy the inhibitory criterion and results in changes in the reinforcement displacement in concrete and eventually creates mechanical cracks and structural destruction. In corrosive environments, due to the corrosion process in the interface and reduction of bonds between rebar and concrete, evaluation and analysis of reinforcement in the concrete according to the mentioned criterion are of great importance. Therefore, the pullout test was conducted on rebar encapsulated in concrete for calculating the existing shear stress, and the deviation from the required value was evaluated. By anticipating the corrosion rate by increasing the effective length of reinforcement or the number of reinforcements used per unit of concrete level [77], it could be understood if the reinforcement would be restrained during the service life span.

The allowable shear stress (proposed for reinforcement by simplifying the governing complex equations and with respect to the experimental coefficients [78]) was calculated using Equation (1).

(1) $$\tau =\frac{\sigma_{y}D}{4l}\;$$

where *D* is the diameter of rebar, $\sigma_{y}$ is the yield stress of steel, *l* is the length of rebar in concrete, and *τ* is the shear stress between rebar and concrete. In the present study, $\sigma_{y}$ = 360 MPa, *D* = 10 mm, and l = 100 mm according to the concrete specimen specifications. Therefore, the allowable shear stress in the rebar and concrete interface is measured as *τ* = 9.25 MPa. To calculate the shear force ($F_{\mathrm{shear}}$), shear stress was multiplied by the effective area of reinforcement in concrete (π. D. l), and $F_{\mathrm{shear}}$ was calculated as 29,045 N. For epoxy coatings, a coefficient of 1.2 was considered [78].

**Figure 9:**
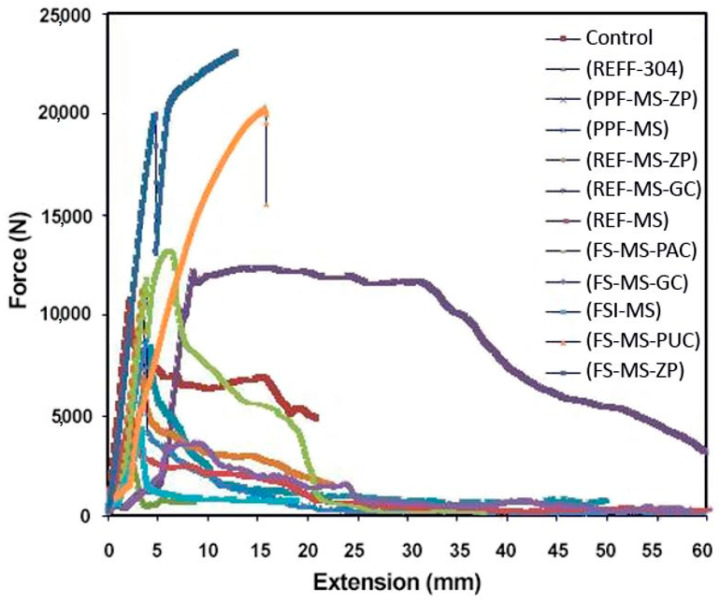
Force versus displacement of selected systems after accelerated corrosion test.

Figure 9 shows the result of the pullout test on steel reinforcement in concrete specimens after the accelerated corrosion test. Due to the limitations, this test was only conducted on non-coated mild steel rebar, AISI 304 rebar, rebars with hot-dip galvanized coating, zinc-rich epoxy primer, and polyurethane top coating.

**Figure 10:**
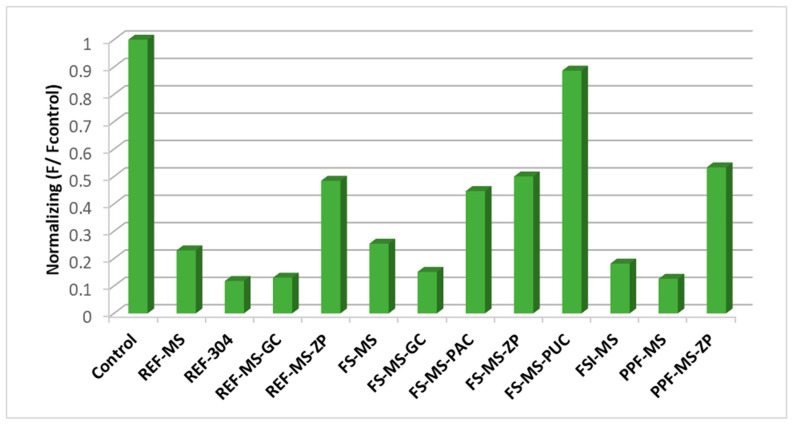
Normalized shear force related to control sample in reinforced concrete in pullout test after 500 h of anodic potential application in 3.5% NaCl solution.

For selected specimens, the amount of shear force, coefficient of application for epoxy coatings, and normalized data compared to the control specimen (non-coated steel reinforcement in the first mix design without corrosion after 14 days of curing) are summarized in Table 12. During the reinforcement pullout test of the concrete samples, the fracture reinforcement (steel yield) was calculated in terms of equilibrium shear stress versus steel yield stress. Figure 10 is used for ease of comparison.

According to Figure 10, zinc-rich epoxy primer and polyurethane top coatings provided the best adhesion to the reinforced concrete because no corrosion occurred in these coatings. AISI 304 rebar had the lowest adhesion after degradation and steel with hot-dip galvanized coating was next in line. The use of Ferrogard 901 inhibitor in S-I-B, compared to S-B sample in the third mix design, led to reducing the reinforcement adhesion to concrete. The decrease in adhesion of hot-dip galvanized reinforcement, due to the reaction on metal and hydration reaction products, was reported in several sources [69,79,80,81]. The bonding between galvanized steel to concrete increases after completing the hydration process due to the formation of calcium hydroxy zinc crystals and filling the porosities in between coating and concrete as a bridge between zinc and concrete coatings [79].

Considering the abovementioned results, the use of Ferrogard 901 inhibitor would not be approved by the structural designer. In the fifth mix design, with the adoption of polypropylene fibers, the non-coated reinforcement had the lowest adhesion to concrete among all samples due to the very severe corrosion of the reinforced concrete. Interestingly, no debonding was observed in the mild steel rebars coated with zinc-rich epoxy primer in concrete with polypropylene fibers. This shows the complete protection of the coating and proper adhesion to the porous mix design. In practice, the bond strength for ribbed steel and galvanized steel is necessarily the same because the bond strength is mainly due to the mechanical locking between the ribbed bumps and concrete [61].

### 4.3. Stereograph

**Figure 11:**
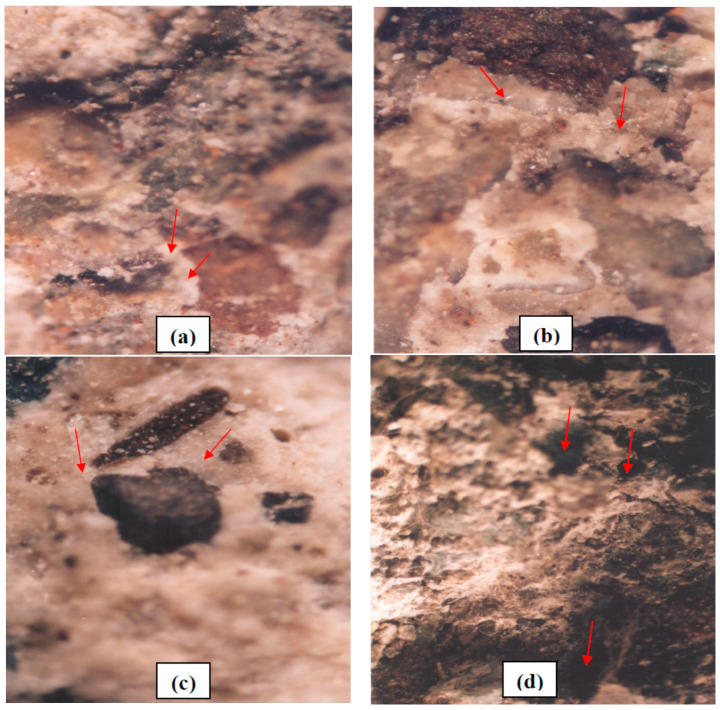
Stereograph microscope images of (**a**) first mix design, (**b**) third mix design, (**c**) fourth mix design, (**d**) fifth mix design (7x magnification).

Stereograph microscope images of the five mix designs are compared in Figure 11. As demonstrated in Figure 11a, showing the first mix design with no additives, the hydrated cement compounds were pervasive around the aggregate. In the third mix design, as provided in Figure 11b, due to the addition of 25% fly ash and 10% silica fume, the compounds surrounding the aggregate (i.e., fly ash, cement, and silica fume) increased and surrounded the aggregates like adhesive. In the fourth mix design, the inclusion of 10% silica fume, 25% fly ash, and 3% Ferrogard 901 inhibitor resulted in increasing the uniformity and better distribution of cement–pozzolanic materials around the aggregates. In general, fly ash and silica fume are also known to have the potential to act as fillers and fill out the micropores of concrete, which in turn reduces the chloride ions’ absorption rate and improves the durability of concrete and rebars. In Figure 11d, polypropylene fibers in the fifth mix design and the air entrapment in the mix design, causing macroscopic cavities in the concrete composition, are shown.

**Figure 12:**
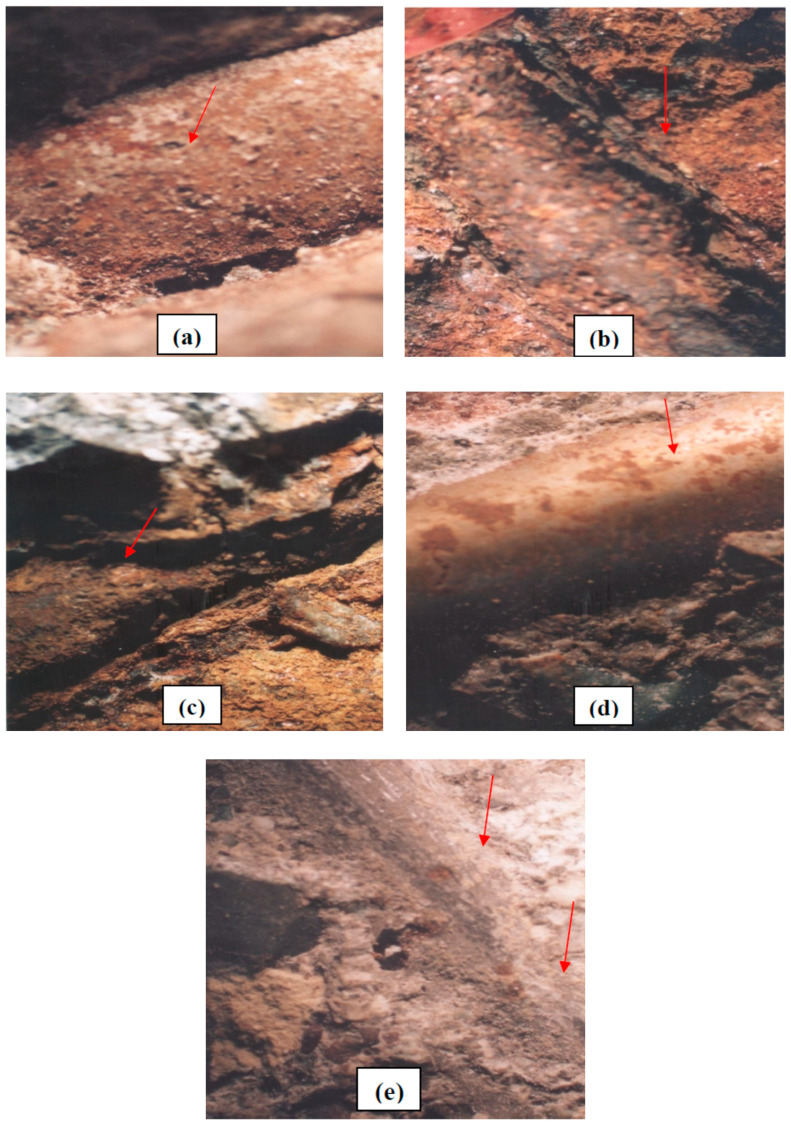
Stereograph microscope images of (**a**) stages of corrosion in non-coated steel reinforcement after 5 h, (**b**) 15 h, (**c**) 25 h, (**d**) AISI 304 rebar after 15 h in the first mix design, and (**e**) zinc-rich epoxy coating steel, after 500 h in the fifth mix design with 32 V potential in accelerated corrosion test (7x magnification).

Figure 12a–c shows the corrosion of non-coated mild steel reinforcement from the breakdown of the oxide layer to the initiation of corrosion and complete destruction after 5, 15, and 25 h of anodic potential. Figure 12d demonstrates the disappearance of the chromium oxide layer after 15 h in AISI 304 rebar compared with non-coated mild steel reinforcement at the same time. Figure 12e also shows the steel reinforcement protection by zinc-rich epoxy primer in the fifth mix design containing polypropylene fibers after 500 h with 32 V potential of anodic. It can be observed that corrosion initiated at low speed (0.003 mpy) in accordance with Table 10, and red iron oxide areas folded over the reinforcement surface are shown.

## 5. Conclusions

This research provides a comprehensive source of information on how to increase the corrosion resistance of rebars by evaluating different concrete mixes, admixtures, rebars, coating systems, and primers. The simultaneous or sole effect of these systems as well as the comparison between their performance by conducting a systematic study provide unique results regarding the selection of the most suitable admixture, rebar, coating system, and primer for application in corrosive environments. The following main conclusions can be derived from the results of this study:

1. Corrosion occurred in the steel-reinforced concrete with a corrosion rate of 56 mpy in accelerated corrosion conditions simulated with 32 V anodic potential. Thus, the first mix design, without the use of concrete additives and with a water/cement ratio of 0.40 and type 2 Portland cement, and the use of non-coated steel reinforcement in environments containing chloride ion is not recommended.
2. The adoption of mild steel reinforcement with hot-dip galvanized coating, AISI 316 rebar, and AISI 304 rebar, due to the existing protective layer, increased the corrosion resistance of reinforced concrete in the first mix design by 2.57, 4.96, and 1.96 times compared to the non-coated mild steel reinforcement, respectively.
3. The inclusion of Ferrogard 901 inhibitor (second mix design) increased the polarization resistance, decreased the corrosion rate by 1.9 times, and significantly increased the structural durability (cracking time) by up to 11.4 times compared to the first mix design.
4. Simultaneous addition of 25% fly ash and 10% silica fume in the third mix design reduced the porosity and increased the corrosion resistance of all protective systems, which substantially decreased the corrosion rate by 1.47 times in the non-coated reinforcement in comparison with the first mix design.
5. The use of the inhibitor delayed the concrete cracking time by up to 1.6 times compared to the adoption of pozzolanic materials.
6. Simultaneous use of 10% silica fume, 25% fly ash, and 3% Ferrogard 901 inhibitor (fourth mix design with type 2 Portland cement and water/cement ratio of 0.40) led to an increase in the cracking time of 11.9 times and a decrease of corrosion rate of non-coated mild steel reinforcement of 2.4 times compared to the first mix design.
7. The adoption of 2.5% polypropylene fibers (vol%) reduced the corrosion rate of steel reinforcement by 2.6 times.
8. After 500 h anodic potential application, polyurethane top coating and zinc-rich epoxy primer with respective values of 0.87 and 0.60 compared to the control sample provided the highest adhesion to the concrete surface.
9. The simultaneous inclusion of silica fume, fly ash, and Ferrogard 901 inhibitor in concrete mix design is recommended for decreasing the porosity of concrete and significantly increasing the corrosion resistance of rebars.

## Figures and Tables

**Table 1:** Chemical compositions of AISI 304, mild steel, and AISI 316 rebars.

Composition	Cr (%)	Mo (%)	Ni (%)	Mn (%) Max	P (%) Max	Si (%) Max	C % (%) Max	S (%) Max
AISI 304	18–20	-	8–10.5	2	0.045	1	0.08	0.03
Mild steel	-	-	-	-	-	-	0.15–0.29	0.03
AISI 316	16-18	2–3	10–14	2	0.045	1	0.08	0.03

**Table 2:** Chemical properties of fly ash, cement, and silica fume [3,35,36,42,43].

Composition	*MgO* (%)	*SO*_3_ (%)	*Al*_2_*O*_3_ (%)	*Fe*_2_*O*_3_ (%)	*CaO* (%)	*SiO*_2_ (%)
Fly ash	3	0.40	30.20	6.50	4.62	51.50
Cement	5.5	1.7	3.30	1.5	62	21
Silica fume	0.10	0.15	0.40	0.70	0.30	94

**Table 3:** Properties of Ferrogard 901 inhibitor [3].

Product	Density (kg/L)	pH	Storage Temperature (°C)	Color/ Appearance	Chemical Composition
Ferrograd 901	1.06	10	35 - 1	Green liquid	2D methyl amino ethanol

**Table 4:** Symbols adopted in this study.

Symbol	Title
REF	First mix design
I	Second mix design
FS	Third mix design
FSI	Fourth mix design
PPF	Fifth mix design
304	Stainless steel AISI 304
316	Stainless steel AISI 316
MS	Non coated mild steel rebar
MS-AC	Mild steel rebar with alkyd top coating
MS-AP	Mild steel rebar coated with alkyd primer (anti-rust)
MS-GC	Mild steel rebar with hot-dip galvanized coating
MS-PUC	Mild steel rebar with polyurethane top coating
MS-PAC	Mild steel rebar with polyamide epoxy top coating
MS-PAP	Mild steel rebar coated with polyamide epoxy primer
MS-ZP	Mild steel rebar coated with zinc-rich epoxy primer
MS-EP-AC	Mild steel rebar with double layers of epoxy primer and alkyd top coating
MS-AP-AC	Mild steel rebar with double layers of alkyd primer and alkyd top coating

**Table 5:** The thickness of primer/coating types [3].

Average Thickness (µm)	Types of Coatings
30 ± 10	MS-AC
220 ± 10	MS-PUC
150 ± 10	MS-EPAC
110 ± 10	MS-APAC
70 ± 10	MS-AP
110 ± 20	MS-ZP
110 ± 10	MS-PAP
230 ± 20	MS-PAC

**Table 6:** Destruction time and corrosion rate of protective systems in the first mix design.

Coating System	Destruction Time (h)	Corrosion Rate (mpy)
REF-MS	26	56.71
REF-MS-GC	67	42.77
REF-304	51	124.78
REF-316	129	158.94
REF-AC	281	16.98
REF-MS-ZP	-	0.002
REF-MS-PAP	-	0.00026
REF-MS-PAC	-	0.00100
REF-MS-PUC	-	0.00015
REF-MS-AC	-	10.55
REF-MS-APAC	-	0.00230
REF-MS-EPAC	-	0.00016

**Table 7:** Destruction time and corrosion rate of protective systems in the second mix design.

Coating System	Destruction Time (h)	Corrosion Rate (mpy)
I-MS	297	19.30
I-MS-GC	324	13.5
I-304	311	12.37
I-316	338	22.61
I-AC	-	0.45
I-MS-ZP	-	0.0010
I-MS-PAP	-	0.035
I-MS-PAC	-	0.063
I-MS-PUC	-	0.000025
I-MS-AC	-	1.44
I-MS-APAC	-	2.25
I-MS-EPAC	-	0.000053

**Table 8:** Destruction time and corrosion rate of protective systems in the third mix design.

Coating System	Destruction Time (h)	Corrosion Rate (mpy)
FS-MS	183	38.53
FS-MS-GC	410	8.008
FS -304	310	28.70
FS -316	316	111.44
FS -AC	-	0.0034
FS -MS-ZP	-	0.0023
FS -MS-PAP	-	0.00032
FS -MS-PAC	-	0.0008
FS -MS-PUC	-	0.00021
FS -MS-AC	-	0.050
FS -MS-APAC	-	0.020
FS -MS-EPAC	-	0.0001

**Table 9:** Destruction time and corrosion rate of protective systems in the fourth mix design.

Coating System	Destruction Time (h)	Corrosion Rate (mpy)
FSI-MS	310	24.025
FSI-MS-GC	-	0.560
FSI-304	-	2.310
FSI-316	350	88.970
REF-AC	-	0.940
FSI-MS-ZP	-	0.011
FSI-MS-PAP	-	0.000023
FSI-MS-PAC	-	0.000043
FSI-MS-PUC	-	0.000056
FSI-MS-AC	-	0.220000
FSI-MS-APAC	-	0.110000
FSI-MS-EPAC	-	0.000014

**Table 10:** Destruction time and corrosion rate of protective systems in the fifth mix design.

Coating System	Destruction Time (h)	Corrosion Rate (mpy)
PPF-MS	10	69.2800
PPF-MS-GC	40	67.9700
PPF-304	11	90.6800
PPF-316	19	176.3400
PPF-AC	333	15.7900
PPF-MS-ZP	-	0.0030
PPF-MS-PAP	-	0.00071
PPF-MS-PAC	-	19.30000
PPF-MS-PUC	-	0.00011
PPF-MS-AC	-	49.92000
PPF-MS-APAC	-	1.05400
PPF-MS-EPAC	-	0.00031

**Table 11:** Time of steel reinforcement demolition in each mix design.

Mix Design	First	Second	Third	Fourth	Fifth
Destruction time (h)	26	297	183	310	10

**Table 12:** Shear force, force correction with experimental coefficients, and normalized numbers relative to control sample force.

Specimen	Force (N)	Force with Coefficient (N)	$\frac{Force}{Force_{control}}$
Control	29,045	29,045	1.00
REF-MS	6251	6251	0.21
REF-304	3148	3148	0.10
REF-MS-GC	3465	3465	0.11
REF-MS-ZP	11,443	13,731	0.47
FS-MS	7012	7012	0.24
FS-MS-GC	3991	3991	0.13
FS-MS-PAC	10,564	12,676	0.43
FS-MS-ZP	11,810	14,172	0.47
FS-MS-PUC	21,187	25,413	0.87
FS-I-B	4876	4876	0.16
PPF-MS	3190	3190	0.51
PPF-MS-ZP	12,564	15,076	0.60

## Data Availability

Some or all data that support the findings of this study are available from the corresponding author upon reasonable request.
